# 3D facial phenotyping

**DOI:** 10.1186/1755-8166-7-S1-I2

**Published:** 2014-01-21

**Authors:** Peter Hammond

**Affiliations:** 1 UCL Institute of Child Health, London, UK

## 

The recognition of facial dysmorphism remains an important skill for clinical geneticists to acquire despite the expanding use of next generation sequencing tools for the identification of gene mutations and related anomalies. Phenotype-genotype correlations, for example, still have an important role to play in diagnosis and prognosis. Facial morphology can now be quickly captured in 3D and analysed with the support of appropriate computer software. This talk will describe how: 3D images of affected individuals can be combined to delineate characteristic facial features of genetic and related conditions; static and dynamic visualisations can help a clinician to identify what is atypical in an individual child’s craniofacial development; quantitative analysis of face shape can assist with diagnosis and the study of phenotype-genotype correlations; links can be established between facial dysmorphism and neuro-cognitive disability arising from genetic anomaly and teratogen exposure.

